# Advancement of the 5-Amino-1-(Carbamoylmethyl)-1H-1,2,3-Triazole-4-Carboxamide Scaffold to Disarm the Bacterial SOS Response

**DOI:** 10.3389/fmicb.2018.02961

**Published:** 2018-12-18

**Authors:** Trevor Selwood, Brian J. Larsen, Charlie Y. Mo, Matthew J. Culyba, Zachary M. Hostetler, Rahul M. Kohli, Allen B. Reitz, Simon D. P. Baugh

**Affiliations:** ^1^Department of Medicine, University of Pennsylvania, Philadelphia, PA, United States; ^2^Department of Biochemistry and Biophysics, University of Pennsylvania, Philadelphia, PA, United States; ^3^Fox Chase Chemical Diversity Center, Inc., Doylestown, PA, United States

**Keywords:** SOS response, antibiotic resistance, structure activity analysis, *Pseudomonas aeruginosa*, DNA damage

## Abstract

Many antibiotics, either directly or indirectly, cause DNA damage thereby activating the bacterial DNA damage (SOS) response. SOS activation results in expression of genes involved in DNA repair and mutagenesis, and the regulation of the SOS response relies on two key proteins, LexA and RecA. Genetic studies have indicated that inactivating the regulatory proteins of this response sensitizes bacteria to antibiotics and slows the appearance of resistance. However, advancement of small molecule inhibitors of the SOS response has lagged, despite their clear promise in addressing the threat of antibiotic resistance. Previously, we had addressed this deficit by performing a high throughput screen of ∼1.8 million compounds that monitored for inhibition of RecA-mediated auto-proteolysis of *Escherichia coli* LexA, the reaction that initiates the SOS response. In this report, the refinement of the 5-amino-1-(carbamoylmethyl)-1H-1,2,3-triazole-4-carboxamide scaffold identified in the screen is detailed. After development of a modular synthesis, a survey of key activity determinants led to the identification of an analog with improved potency and increased breadth, targeting auto-proteolysis of LexA from both *E. coli* and *Pseudomonas aeruginosa*. Comparison of the structure of this compound to those of others in the series suggests structural features that may be required for activity and cross-species breadth. In addition, the feasibility of small molecule modulation of the SOS response was demonstrated *in vivo* by the suppression of the appearance of resistance. These structure activity relationships thus represent an important step toward producing Drugs that Inhibit SOS Activation to Repress Mechanisms Enabling Resistance (DISARMERs).

## Introduction

Antibiotic resistant bacteria represent one of the most pressing issues in infectious disease research today (Brown and Wright, 2016). An era is fast approaching when many currently treatable infections may become incurable (Boucher et al., 2009). While important efforts are underway to discover antimicrobials with different mechanisms of action (Clatworthy et al., 2007; Thaker et al., 2013; Ling et al., 2015), the most conventional approach to overcoming resistance has involved the chemical modification of existing antibiotic scaffolds (Fischbach and Walsh, 2009). Although the resulting “next generation” antibiotics offer a respite, bacteria are likely to rapidly adapt their preexisting resistance mechanisms to counteract these gains. The limitations of conventional approaches highlight the need to pursue alternative strategies.

**FIGURE 1:**
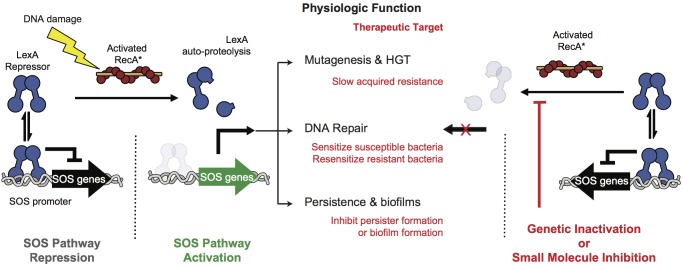
SOS pathway activation and inhibition. At left, under non-stressed conditions the intact LexA dimer binds to the SOS promoter region and tightly represses SOS protein production. Upon DNA damage RecA binds to DNA fragments to form RecA^∗^ filaments that stimulate free LexA to undergo auto-proteolysis. Auto-proteolysis of LexA renders it unable to bind to the SOS promoters leading to expression of the effectors of the SOS pathway. These effectors promote DNA repair, accelerate gene transfer and mutagenesis, and contribute to virulence. At right, genetic inhibition of the SOS response has been shown to antagonize these antibiotic-evasion associated phenotypes and small molecule inhibition of the pathway is therefore a viable pathway for improving the efficacy of antibiotics or targeting virulence.

A promising alternative approach is to target pathways that promote acquired resistance to antibiotics. One such pathway is the bacterial DNA damage response pathway, known as the SOS response (Figure 1). Many antibiotics induce the SOS response, either by inducing DNA damage (e.g., fluoroquinolones) or by indirectly promoting DNA damage via targeting essential cellular and metabolic functions (Kohanski et al., 2007; Dwyer et al., 2012; Mo et al., 2016). The SOS response is well conserved across pathogens and involves numerous genes (e.g., >40 in *Escherichia coli*). These proteins include translesion DNA polymerases that promote mutagenesis, recombinases that mobilize antibiotic resistance genes, and proteins that mediate persistence, biofilm formation or directly promote antibiotic evasion (McKenzie et al., 2000; Beaber et al., 2004; Schlacher et al., 2006; Galhardo et al., 2007; Da Re et al., 2009; Dörr et al., 2009, 2010; Gotoh et al., 2010). Thus, suppression of the SOS pathway would be predicted to compromise the response of bacteria to antibiotics.

A means to suppress the SOS pathway is to maintain repression of the SOS response. In the absence of genotoxic stress all genes of the pathway are tightly repressed by the dual-function repressor/protease, LexA (Figure 1). In the presence of genotoxic stress the DNA damage sensor protein RecA forms filaments along ssDNA generated by aborted replication. The pathway is triggered when this filamentous RecA (RecA^∗^) promotes a conformational change in LexA that brings one of its protein loops into its own serine protease active site (Luo et al., 2001). Subsequent auto-proteolysis destabilizes LexA, and leads to transcriptional de-repression of SOS pathway genes (Culyba et al., 2018).

Genetic studies targeting either RecA or LexA validate the SOS response as a therapeutic target (Figure 1). In a murine thigh infection model an *E. coli* strain harboring a non-cleavable mutant of LexA abrogated resistance both to ciprofloxacin and rifampicin compared to a strain with a cleavable LexA (Cirz et al., 2005). In addition, deletion of RecA, or forced over expression of non-cleavable LexA have been shown to hyper-sensitize bacteria to traditional antibiotics (Lu and Collins, 2009; Thi et al., 2011; Mo et al., 2016). Furthermore, SOS inactivation in resistant bacteria resulted in re-sensitization to a fluoroquinolone (Recacha et al., 2017). Together, these studies suggest that targeting the SOS response could lead to both synergy with DNA damaging antibiotics to lower MIC values and suppression of acquired resistance (Cirz and Romesberg, 2007; Smith and Romesberg, 2007; Culyba et al., 2015).

**FIGURE 2:**
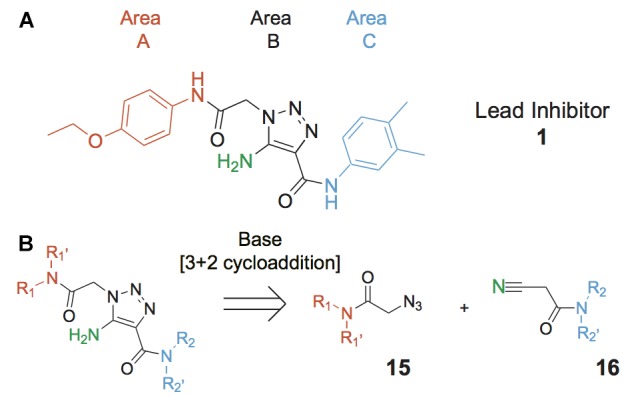
Lead compound and synthetic approach. **(A)** The lead **1** is shown with the Areas A, B and C highlighted. These areas are the focus of diversification in analog synthesis to explore structure-function relationships in the lead series. **(B)** Retrosynthesis of the 5-amino-1-(carbamoylmethyl)-1H-1,2,3-triazole-4-carboxamides is shown, with the core of Area B formed via a cycloaddition of azide **15** and nitrile **16**. In Area B the 5-amino group derived from the nitrile is highlighted to help illustrate the cycloaddition mechanism.

While specifically targeting RecA has produced some important gains (Wigle et al., 2009; Alam et al., 2016; Bellio et al., 2017), we aimed to inhibit the RecA^∗^-induced cleavage of LexA as this represents the key initiating step in the SOS response. To this end we developed a high throughput screen (HTS) that allowed estimation of RecA^∗^-mediated LexA cleavage. Using this screen some 1.8 million compounds were evaluated for inhibition of RecA^∗^-mediated LexA cleavage (Mo et al., 2018). The result of this screen was the identification of several chemotypes with the potential to modulate the SOS response (Mo et al., 2018). Herein is described the advancement of one of the chemotypes, the 5-amino-1-(carbamoylmethyl)-1H-1,2,3-triazole-4-carboxamide scaffold (Figure 2) via a modular synthesis that allowed for evaluation of structure-activity relationships and lead improvement to increase potency and expand the breadth of targetable pathogens. This work underscores the feasibility of developing DISARMERs (Drugs to Inhibit SOS Activation to Repress Mechanisms Enabling Resistance) – molecules that can act as adjuvants in standard antimicrobial therapies to both sensitize bacteria to antibiotics and reduce the rise of acquired resistance.

## Materials and Methods

### Materials

All reagents used in chemical synthesis were purchased from Aldrich Chemical Co., (Milwaukee, WI, United States), Alfa Aesar (Ward Hill, MA, United States), or Thermo Fisher Scientific (Pittsburgh, PA, United States) and were used without further purification. Chemicals used in biochemical assays were obtained from Sigma-Aldrich (St Louis, MO, United States).

### Compound Synthesis

Compounds were synthesized using a method that proceeds via a [3+2] cycloaddition, allowing facile, catalytic, non-moisture sensitive, and non-air sensitive syntheses of a variety of 5-amino-1-(carbamoylmethyl)-1H-1,2,3-triazole-4-carboxamides. For the majority of analogs, catalysts employed were either sodium ethoxide (synthesis A, Table 1) or cesium carbonate (synthesis B, Table 1). The base-mediated cyclization is depicted in Figure 2.

**Table 1:** Synthesis and inhibition by lead analogs.

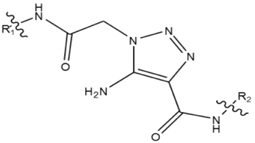					

**Compound**	**R_1_**	**R_2_**	***E. coli*** **IC_50_/μM^1^**	**Synthesis^2^**	**Yield%**

**1**	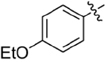	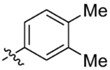	32 ± 6	**A/B**	25/25
**2**	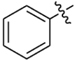	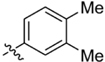	>100	**n/a**	commercial
**3**	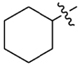	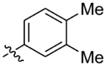	44 ± 4	**A**	27
**4**	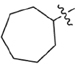	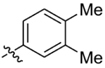	22 ± 3	**B**	34
**5**	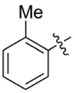	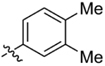	17 ± 2	**n/a**	commercial
**6**	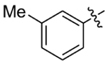	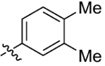	>100	**n/a**	commercial
**7**	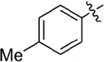	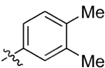	18 ± 1	**C**	21
**8**	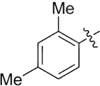	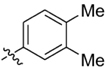	15 ± 2	**B**	31
**9**	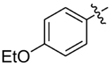	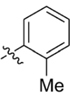	>100	**n/a**	commercial
**10**	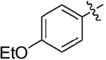	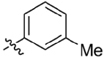	33 ± 4	**A**	32
**11**	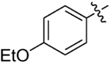	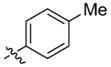	15 ± 2	**n/a**	commercial
**12**	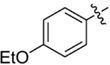	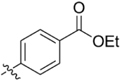	19 ± 3	**B**	12
**13**	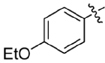	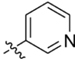	40 ± 3	**B**	33
**14**	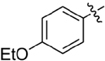	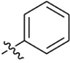	9 ± 1	**B**	39


*^*1*^IC50 values are the avearge of 4 determinations and the errors are ± 1 SD. ^*2*^A-NaOEt conditions. B-CS2CO3 conditions, C-NaOMe conditions.*

For reactions catalyzed by sodium ethoxide (synthesis A), a solution of sodium ethoxide (1.2 mmol) in anhydrous ethanol (10 mL) was maintained under nitrogen and cooled to 0°C with stirring. Once cooled, the cyano component (1.1 mmol) was added to the solution. The resulting solution was stirred for 10 min at 0°C before addition of the azido component (1.0 mmol). The resulting solution was maintained at 0°C for a further 2 min before being allowed to warm to room temperature. Upon reaching room temperature, the solution was lightly sonicated for 20 s before the temperature was raised to 40°C. The solution was maintained at 40°C for 4 h before being allowed to cool to room temperature. Once the solution reached room temperature the reaction was quenched with deionized H_2_O (100 mL) and extracted with ethyl acetate (3 × 50 mL). The combined organic fractions were washed with deionized H_2_O (50 mL), dried over anhydrous Na_2_SO_4_, filtered, and concentrated in vacuo to yield the crude product. The crude product was dissolved in DMF, filtered through a syringe filter and purified via reverse phase HPLC using acetonitrile in deionized H_2_O (with 0.1% TFA in both solvents) to yield, after evaporation and lyophilization, the desired product.

For reactions catalyzed by cesium carbonate (synthesis B), the azido component (1.1 mmol), the cyano component (1.0 mmol) and cesium carbonate (0.25 mmol) were dissolved in DMSO/deionized H_2_O (7:3, 4 mL) with stirring. The reaction vial was capped and stirred for 24 h before being diluted with deionized H_2_O, partially concentrated in vacuo, frozen, and lyophilized to remove water and DMSO. The resulting crude material was purified via reverse phase HPLC using acetonitrile in deionized H_2_O (with 0.1% TFA in both solvents) to yield, after evaporation and lyophilization, the desired product.

For compound **7** a variation of synthesis A (synthesis C) was employed in which sodium methoxide was used instead of sodium ethoxide. For this synthesis a mixture of the cyano component (0.24 mmol), and sodium methoxide (0.26 mmol) in methanol (1.07 mL) was stirred for 30 min before addition of the azido component (0.21 mmol). The reaction was stirred for 16 h before treatment with methanol (0.25 mL) and stirring for 3 h. Methanol (1 mL) was added, and the reaction was heated at 95°C for 2 h. The mixture was treated with deionized water (25 mL), concentrated HCl (1 drop), and ethyl acetate (10 mL). The aqueous layer was treated with saturated aqueous NaHCO_3_ (5 mL) and extracted with ethyl acetate (10 mL). The combined organic extracts were washed with saturated aqueous NaHCO_3_ (10 mL), brine (10 mL), and dried over anhydrous MgSO_4_ before concentration. The crude material was purified via reverse phase HPLC using acetonitrile in deionized H_2_O (with 0.1% TFA in both solvents) to yield, after evaporation and lyophilization, the desired product.

Some compounds in the Supplementary Information were synthesized by alternative methods in which an acetylene component replaced the cyano component. The alternate syntheses are described in the Supplementary Methods. Analogs which were not synthesized were obtained from commercial vendors ChemDiv (San Diego, CA, United States) and Vitas-M Laboratory (Champagne, IL).

All compounds were readily soluble in DMSO and were stored as 10 mM frozen (-30°C) stocks when not in use.

### FlAsH-LexA Cleavage Assay

IC_50_ values were routinely determined using the *E. coli* FlAsH-LexA cleavage assay previously used to perform HTS (Mo et al., 2018). In this assay RecA-promoted LexA cleavage is monitored using fluorescence polarization. The *E. coli* FlAsH-LexA and RecA were constructed, expressed and purified as previously described (Mo et al., 2018). The conditions were 100 nM *E. coli* FlAsH-LexA, 200 nM RecA, 5 μM ssDNA (SKBT25: GCG TGT GTG GTG GTG TGC) (Tracy and Kowalczykowski, 1996), 5 μM ATPγS in 100 mM Tris-HCl, pH 6.5, 150 mM NaCl, 5 mM MgCl_2_, 0.1 mM TCEP, and 0.01% (w/v) Pluronic-F127. Reactions were performed in 384-well plates and components were added as 10 μL additions of ATPγS, ssDNA and RecA, in buffer and 10 μL of *E. coli* FlAsH-LexA in buffer using a Janus liquid handler (Perkin-Elmer). Compound was added as a DMSO solution using a pin tool, and the final concentration of DMSO in the reaction was 1.2%. Once the reaction components were combined, reactions were centrifuged for 1 min at 500 rpm and incubated for 30 min at room temperature. Reactions were quenched with a 10 μL addition of 50 mM EDTA and plates were read on a Tecan Infinite F200 Pro plate reader (Tecan US, Inc., Morrisville, NC, United States). The final assay conditions resulted in 100–120 mP difference between the uncleaved and cleaved control wells, representing an approximately 60% cleavage of the *E. coli* FlAsH-LexA. On each plate 32 positive (-RecA) and 32 negative controls (+RecA) in which DMSO without compound was added were used to define the range of mP and calculate the fraction inhibited.

IC_50_ values were estimated by non-linear least squares fitting to the data using Equation 1.

(1) $$\mathrm{FI}=\frac{{\left[I\right]}^{n}}{{\mathrm{IC}}_{50}^{n}+I^{n}}$$

where FI = Fraction inhibited, [I] = Concentration of compound and *n* = Hill coefficient. Fitting was performed using Igor Pro (WaveMetrics Inc., Lake Oswego, OR, United States).

In the FlAsH-LexA cleavage assay the highest compound concentration was 111 μM and all of the compounds that demonstrated activity (**1**, **3**, **4**, **5**, **7**, **8**, **10**–**14**, **22,** and **23**) elicited normal titration curves suggesting that aqueous solubility was maintained up to 111 μM. Representative titrations for the compounds can be found in the Supplementary Figure 1.

### Orthogonal ^32^P-LexA Cleavage Assay

Full-length *E. coli* and *P. aeruginosa* LexA were engineered with a RRXS phosphorylation site on the N-terminus of the full-length protein, allowing for ^32^P labeling by protein kinase A to produce ^32^P-LexA, as described previously (Mo et al., 2018). Reactions contained 100 nM ^32^P-LexA, 200 nM RecA and 10 μM ATPγS and the buffer conditions were identical to those in the HTS assay. Compounds were added in DMSO and the final concentration was 2%. Reactions were incubated for 30 min at room temperature after which 2 × Laemmli buffer was added to stop the assay. The stopped reactions were subjected to 15% SDS-PAGE and the gels were visualized via phosphorimaging on a Typhoon imager (GE Healthcare Bio-Sciences, Marlborough, MA, United States). The intact and cleaved bands were quantified using Quantity One (Bio-Rad, Hercules, CA, United States) and the fraction inhibited was calculated. As for the HTS assay, controls contained DMSO and the negative controls contained RecA while the positive controls did not. Plots of fraction inhibited against compound concentration were fitted to Equation 1.

### Electrophoretic Mobility Shift Assay

For the electrophoretic mobility shift assay (EMSA) full-length, catalytically inactive LexA-S119A was used (Mo et al., 2014). Increasing concentrations (0–1 μM) of LexA-S119A were mixed with 10 nM SOS operator DNA labeled with Cy5 in EMSA running buffer (70 mM Tris-HCl pH 7.5, 10 mM MgCl_2_, 150 mM NaCl, 5 mM DTT, 0.1 mg/ml BSA, 10 ng/μL ssDNA, 5% glycerol, 0.04% bromophenol blue) in the presence of 50 μM of compound (or DMSO carrier). After incubation at room temperature for 30 min, 20 μL of each reaction was subjected to 6% native PAGE. Gels were visualized on a Typhoon Imager using default fluorescence filter settings for Cy5. Gel bands were quantified using ImageJ (NIH, Bethesda, MD, United States) to determine the fraction of bound DNA at each LexA concentration. Data were fitted to a variable-slope sigmoidal dose-response curve.

### Cell-Based SOS Reporter Assay

An *E. coli* MG1655 strain lacking *sulA* (Δ*sulA*) and the *tolC* transporter (Δ*tolC*) (Mo et al., 2016) was transformed with a reporter plasmid in which *gfp* expression was under the control of the *recA* promoter (pMS201 p*RecA* GFP) (Zaslaver et al., 2006). To perform assays overnight, cultures of the reporter strain were diluted 100-fold in M9 minimal media and grown at 37°C with agitation to an OD_595_ of ∼0.6. For each reaction sample 100 μL of culture were mixed with 100 μL of M9 minimal media containing ciprofloxacin (256 ng/mL). Pre-diluted compounds were added (5 μL) in DMSO and cultures were incubated at 37°C with agitation for 2 h after which the cells were fixed by adding 200 μL of phosphate buffered saline, pH 7.4 containing 1% paraformaldehyde. After 1 h of fixing, the cells were spun down at 4,000 rpm and re-suspended in phosphate buffered saline, pH 7.4. Fixed cells were analyzed using flow cytometry (BD FACSCalibur, Ex/Em: 488 nm/530 nm) and the mean fluorescence of 20,000 cells in each condition was recorded.

### Frequency of Resistance

A starter culture of Δ*tolC E. coli* was cultured overnight at 37°C with shaking in LB broth. The next day the culture was diluted 3 × 10^7^-fold in to LB broth. The dilution was used to produce four sets of twelve cultures, each containing 1 mL. To one set was added 10 μL of deionized H_2_O plus 10 μL of DMSO, to the second set was added 10 μL of 125 ng/mL ciprofloxacin in deionized H_2_O plus 10 μL of DMSO, to the third set was added 10 μL of deionized H_2_O plus 10 μL of a 10 mM solution of **14** in DMSO and to the fourth set was added 10 μL of 125 ng/mL ciprofloxacin in deionized H_2_O plus 10 μL of **14** in DMSO. The final concentration of ciprofloxacin (1.25 ng/mL) was below the MIC for ciprofloxacin which was determined to be 5 ng/mL.

The 48 cultures were incubated at 37°C with shaking for 48 h. To determine the population size, spot plating was performed starting with 1 μL of the cultures diluted 10^5^-fold. A 100 μL aliquot of each 10^5^-fold dilution was transferred to a 96-well plate and serially diluted (10-fold dilutions) into LB broth. The dilutions (5 μL) were spotted on LB agar plates and the plates were incubated overnight at 37°C. To determine the rifampin resistant population, 999 μL of each 1 mL culture was centrifuged at 6000 rpm for 10 min to remove the cells from solution and the cells were suspended in 100 μL of autoclaved 0.15 M NaCl. The 100 μL solutions were plated on the LB plates containing 100 μg/mL rifampin and incubated for 2 days at 37°C. Following counting of the colonies the program bz-rates (Gillet-Markowska et al., 2015) was used to estimate mutation rates.

## Results

Among the leads isolated from the HTS performed for inhibitors of RecA^∗^-mediated LexA cleavage, lead **1** was selected for progression (Figure 2A). In the initial HTS, the parent 5-amino-1-(carbamoylmethyl)-1H-1,2,3-triazole-4-carboxamide, **1**, had an IC_50_ value of 32 μM (Table 1). This chemotype was well behaved in the HTS, producing close to 100% inhibition, and appeared to offer the most chemical tractability to allow for the construction of structure activity relationships (SARs). Furthermore, as LexA cleavage involves formation of a β-turn at the site of self-cleavage (Lee et al., 2005; Whitby et al., 2011), the structural similarity of **1** to β-turn mimetics also suggested that structure-activity relationships could inform on the possible mode of inhibitor action.

In order to better understand SARs, a modular synthesis was devised that would permit generation of informative analogs. While the construction of 5-amino-1,4-disubstituted-1,2,3-triazoles has been extensively investigated (Tome, 2004) no synthetic routes to 5-amino-1-(carbamoylmethyl)-1*H*-1,2,3-triazole-4-carboxamides based on **1** have yet been reported.

Initial synthetic routes that proceeded via the generation of two potential carboxylic acid intermediates followed by peptide couplings to vary the left- and right-hand portions of the final product were unsuccessful. These reactions were low yielding and/or the precursors were prone to decomposition. A more successful strategy proved to be to proceed via the simple structural intermediates, azides (**15**) for the left-hand portion and nitriles (**16**) for the right-hand portion (Figure 2B). These intermediates were either synthesized in 1–2 steps (Hering et al., 2005; Ju et al., 2006; Srinivasan et al., 2006; Ng et al., 2008; Xia et al., 2014) or purchased directly and could be combined via known base-mediated conditions to produce the desired aminotriazoles via a [3+2] cycloaddition.

Three sets of reagents that have been previously reported to facilitate such cyclizations were screened: stoichiometric sodium methoxide (Alfred, 1970; L’abbé and and Beenaerts, 1989; Julino and Stevens, 1998), stoichiometric sodium ethoxide (Hoover and Day, 1956; Livi et al., 1979), and catalytic cesium carbonate (Krishna et al., 2015). In most cases the choice of base between stoichiometric sodium ethoxide and catalytic cesium carbonate had little to no impact on the yield (e.g., **1**, Table 1). Overall most reactions were successful using the cesium carbonate conditions, however, yields using this route were affected by the time and temperature of the reaction. Using either the sodium ethoxide or catalytic cesium carbonate routes readily permitted modular access to a large variety of analogs, as demonstrated by the fact that aromatic, heteroaromatic, and non-aromatic groups for R_1_ and R_2_ were tolerated (Table 1). This modular approach thus allowed for systematic variation and investigation of structure activity relationships.

### Structure Activity Relationships

Initial medicinal chemistry efforts focused on developing an understanding of the necessary features to improve potency. The three areas (A, B, and C) in Figure 2A were systematically investigated and the IC_50_ values for selected compounds are listed in Table 1 with additional data shown in Supplementary Table 1. Approaches used to probe the binding of compounds of this class included amino group replacement, linker methylation and N-methylation, methyl probing of the aryl rings, homologated variations, and non-aromatic variations. IC_50_ values were determined using the FlAsH-LexA cleavage assay.

In the linker connecting areas A and B, both mono- and bis-methylated compounds (**17** and **18**) showed no measurable activity, suggesting that substitution was not tolerated at the methylene linker. Similarly, methylation of the amide of the linker, **19**, also abrogated activity. The inability to tolerate substitution in the linker region suggested that it likely lies in a narrow groove and that attempts to modify this area could impact the conformations accessed by the lead. With the linker area appearing not amenable to modification, the aromatic portion of area A was investigated. Replacement of the para-ethoxy group substituted phenyl ring with an unsubstituted phenyl, **2**, benzyl, **20**, or phenethyl group, **21**, led to loss of activity. However, replacement of the phenyl ring with a cyclohexyl ring, **3**, or a cycloheptyl ring, **4**, returned activity, suggesting that aromaticity was a larger restriction than hydrophobicity. Systematic variation of methyl functionalization on the phenyl ring revealed that substitution at the meta position, **6**, was not tolerated whereas substitution on the ortho, **5**, and para, **7**, positions were preferred. Beyond the single methyl functionalization, mono-substitution at the ortho position on the phenyl showed steric preferences with activities: Me (**5**) > Et (**22**) > OMe (**23)** > OEt (**24**) = H (**2**). Bis-substitution on the aryl ring was additionally investigated with for example, **8**, showing that combination of ortho- and para-substitution was tolerated but not significantly superior to ortho-substitution alone, **5**. In summary, probing of area A revealed interesting substrate preferences but failed to produce a significant increase in potency.

Additional investigations in area B also did not reveal a means to increase potency. Replacement of the amine by hydrogen (**25**), methyl (**26**), or ethyl (**27**) all rendered compounds inactive, suggesting that the amine was making contacts essential for activity. Supporting this conclusion was the finding that mono-substitution on the amine by acetyl (**28**) was tolerated but with reduced potency.

Probing of area C proved more fruitful. As with area A, the amide linker appeared important as methylation was not tolerated (**29**). Probing of the phenyl moiety indicated that its presence and correct positioning are critical. The importance of this ring was indicated by the intolerance to replacement by cyclohexyl (**30**) or methyl (**31**) groups, and the need for correct positioning was indicated by the intolerance to the replacement of the phenyl ring by benzyl (**32**) or phenethyl (**33**). The importance of substitution on the phenyl ring was investigated by systematic methyl substitution around the phenyl ring. Consistent with the meta- and para- substitution pattern on area C of **1**, this analysis indicated that ortho substitution, **9**, was not tolerated whereas individual meta, **10**, and para, **11**, substitutions were allowed. Heteroatom inclusion was also tolerated in Area C, as shown by ester-containing variation **12**, and pyridyl derivative **13**. However, compound **14**, 5-amino-1-{2-[(4-ethoxyphenyl)amino]-2-oxoethyl}-N-phenyl-1H-1,2,3-triazole-4-carboxamide, with no substitution on the phenyl ring proved to be the most potent compound tested in this series with an IC_50_ of 9 μM.

Before proceeding to additional analysis, cytotoxicity testing with HG2 cells was performed for select compounds, including **1** and **14**. Both the initial lead **1** and the most potent analog **14** showed no appreciable toxicity (CC_50_ of 277 μM and > 500 μM, respectively). Due to the increased potency and lack of cytotoxicity, the mechanism and activity of compound **14** was examined in more detail as described below.

### Characteristics of Compound 14

**FIGURE 3:**
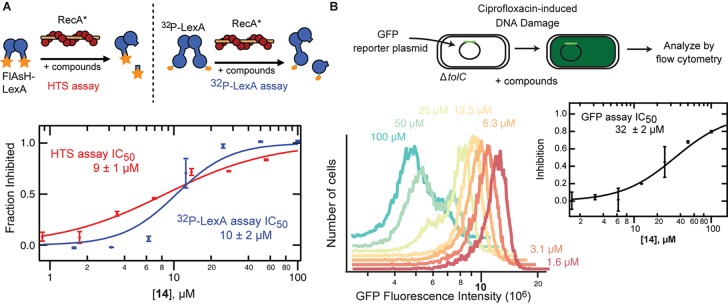
Validation of *in vitro* and *in vivo* activity of **14**. **(A)** The complete dose response curve for **14** performed using two orthogonal *in vitro* assays are shown. The HTS assay employs RecA^∗^-induced changes in fluorescence polarization that can be tracked upon proteolysis of a fluorescent, truncated version of LexA. The ^32^P-LexA assay uses N-terminal ^32^P-labeled LexA and tracks formation of the N-terminal fragment upon RecA^∗^-induced cleavage. The calculated IC_50_ values are shown with standard deviation from at least three replicates. **(B)** The SOS reporter assay employs a plasmid with GFP downstream of a *recA* promoter. Expression of GFP can be tracked after initiating DNA damage with ciprofloxacin in a Δ*tolC* MG1655 *E. coli* strain. At left, flow cytometry plots from a representative experiment are shown as a density plot showing the level of GFP expression in the presence of serial dilutions of **14**. At right, the mean GFP fluorescence was used to calculate the level of inhibition relative to a negative control in the absence of **14** and a positive control in the absence of DNA damage.

A suite of assays was utilized to examine **14** in order to confirm specific inhibition against LexA and demonstrate SOS suppression in cells. To confirm the findings from the FlAsH-LexA cleavage assay, a fluorescence-independent assay using a full-length version of *E. coli* LexA was employed. The full-length LexA contained a PKA phosphorylation site at the N-terminus that allowed ^32^P labeling, such that the extent of auto-proteolysis can be visualized by phosphor-imaging following SDS-PAGE. A plot of the titration curve of **14** obtained using this methodology is shown along with a titration obtained using the FlAsH-LexA assay in Figure 3A. The IC_50_ of 10 ± 1 μM indicates that the IC_50_ obtained using the fluorescently labeled truncated *E. coli* LexA in the HTS assay (9 ± 1 μM) was not due to a fluorescence artifact, and that similar potency is observed with full-length and truncated LexA. Interestingly, a similar IC_50_ value was obtained when the slow cleavage in the absence of RecA^∗^ was monitored (Supplementary Figure 2A). This suggests that **14** binds specifically to LexA, which is further supported by the observation of a thermal shift assay of LexA in the presence of **14** (Supplementary Figure 2B).

The dual activities of LexA, DNA binding and protease activity, permit confirmation of specificity. If **14** inhibits RecA^∗^-mediated LexA cleavage in the expected manner, it would be predicted to inhibit the protease function of LexA, but not to alter DNA binding. To examine LexA binding to DNA in the presence of **14** an EMSA was employed. As with **1** (Mo et al., 2018), LexA showed similar DNA binding affinity in the presence or absence of **14** (Supplementary Figure 3). This observation confirms that the effects in the HTS and ^32^P-LexA assays are not due to non-specific aggregation of LexA or other artifacts. Another important consideration is the permeability of **14** in to the bacteria. Permeability was assessed using a Δ*tolC* strain of *E. coli* containing a plasmid that contained the GFP gene under the control of the *recA* promotor (Mo et al., 2018). Compound **14** inhibited the appearance of GFP fluorescence in a dose-dependent manner with an IC_50_ value of 32 ± 2 μM indicating permeability into the Δ*tolC* strain of *E. coli* (Figure 3B), without impacting cell size (Supplementary Figure 4). The less potent value compared to *in vitro* values suggests that even in the efflux-compromised *E. coli* strain there still remain barriers to entry.

**FIGURE 4:**
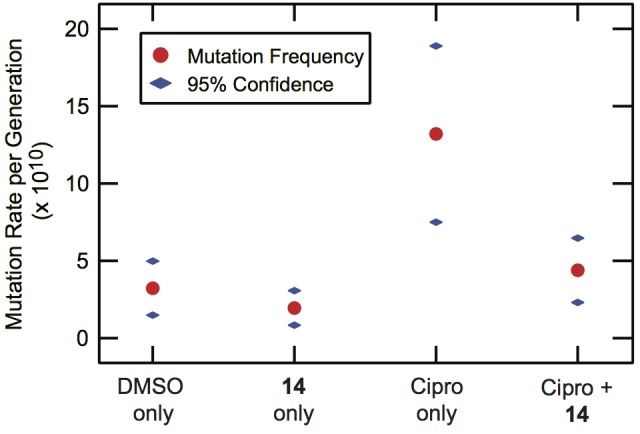
Suppression of ciprofloxacin-induced mutagenesis by **14**. Δ*tolC* MG1655 *E. coli* cultures were grown in the presence or absence of **14** (100 μM) and/or a sub-MIC level of ciprofloxacin (1.25 ng/mL). The cultures were plated without selection to determine total population size and on selective rifampin-containing media (100 μg/mL) to quantify the frequency of rifampin-resistance in the population. The mutational frequency was converted to a per-generation mutation rate, with the rate and 95% confidence interval shown. The rate data were calculated based on at least twelve independent cultures under each condition.

Although the permeability remains in need of further improvement, we also examined whether **14** could suppress the downstream effects of the SOS response *in vivo*. With the knowledge that the IC_50_ for permeability in the Δ*tolC* strain of *E. coli* was 32 ± 2 μM, a concentration of 100 μM **14** was used to assess the ability of **14** to suppress the ciprofloxacin-induced appearance of resistance to rifampicin. As can be seen from Figure 4, the lead **14** was effective in reducing the appearance of resistance to rifampicin. In the presence of **14** alone, the mutation rates were comparable to DMSO alone controls. Conversely, exposure to a sub-MIC concentration (1.25 ng/mL) of ciprofloxacin produced an induction of mutagenesis. In the presence of ciprofloxacin and **14** together, an approximately threefold decrease in the per generation mutation rate was observed relative to ciprofloxacin alone.

### Cross-Species Reactivity

**FIGURE 5:**
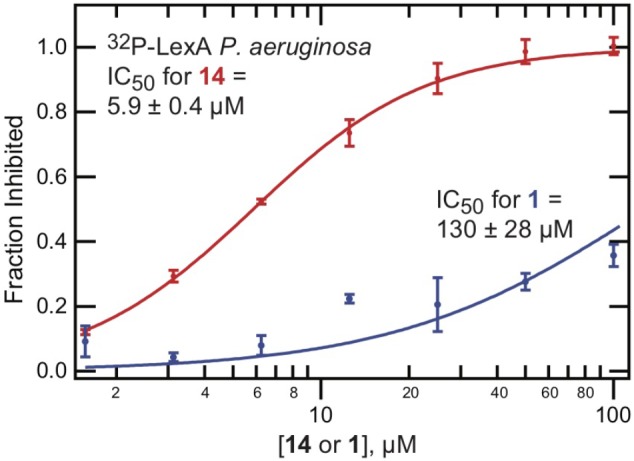
Improved cross-species activity of **14**. The ^32^P-LexA assay, examining RecA^∗^-mediated cleavage of full length LexA from *P. aeruginosa*, was performed using serial dilutions of either **1** or **14**. The percent inhibition was calculated relative to DMSO controls. The mean value is shown with standard deviation, calculated from at least two replicates.

The HTS and medicinal chemistry efforts were directed at the inhibition of *E. coli* LexA auto-proteolysis. To determine the extent of cross-species reactivity, the effectiveness of **14** in inhibiting the RecA-promoted auto-proteolysis of *Pseudomonas aeruginosa* LexA was examined. As can be seen from Table 1 and Figure 5, compound **14** inhibited the RecA-mediated auto-proteolysis of *P. aeruginosa* LexA with similar potency (IC_50_ = 5.9 ± 0.4 μM) to that demonstrated with full-length *E. coli* LexA (IC_50_ = 10 ± 1 μM). This behavior was not observed with **1** (Figure 5) which was a less potent inhibitor of RecA^∗^-induced auto-proteolysis of full-length *P. aeruginosa* LexA (IC_50_ = 130 ± 28 μM). Thus, minor modifications to **14** compared to **1** had a significant effect on cross-species reactivity and permits potentially expanded species breadth.

## Discussion

While the HTS for inhibitors of RecA^∗^-mediated LexA cleavage produced several chemotypes, the 5-amino-1-(carbamoylmethyl)-1H-1,2,3-triazole-4-carboxamide scaffold appeared the most amenable for advancement. The low cytotoxicity and the β-turn mimetic-like structure (see below) were important considerations in the choice to advance this chemotype. A particularly important consideration was the chemical tractability of the lead compound, which permitted the development of a highly modular synthesis that allowed for an initial survey of structure-activity relationships. Our synthetic approach is important because compounds containing the privileged 5-amino-1-(carbamoylmethyl)-1H-1,2,3-triazole-4-carboxamide scaffold have been used to target broad categories of biological activity. Targets have included C3d of the immune response (Morikis and Gorham, 2016), *Mycobacterium tuberculosis* proteasome (Mehra et al., 2015, 2016), microRNA for the treatment of certain cancers (Calin et al., 2002), and a wide range of other diseases (Tili et al., 2007; Huang et al., 2010, 2012, 2013).

Inhibition of the SOS response can now be added to the list of uses for the 5-amino-1-(carbamoylmethyl)-1H-1,2,3-triazole-4-carboxamide scaffold. More specifically, the similar IC_50_ values for **14** in the fluorescence-based LexA cleavage assay and an orthogonal ^32^P-LexA ± RecA assay suggests on-target activity. This effect appears specific for the self-cleavage activity of LexA, because EMSA testing indicated that **14** does not interfere with the DNA binding ability of LexA. The fact that one LexA function is inhibited while the other is preserved further suggests that **14** is not a Pan-Assay Interference (PAINS) inhibitor (Aldrich et al., 2017). While the data suggest **14** binds specifically, the exact binding site is not clear. We have previously speculated that a β-turn mimetic may prove a useful strategy for targeting the LexA active site given that a β-turn formation plays a role in self-cleavage (Mo et al., 2016). Indeed, speculation that this scaffold could function as a β-turn mimetic was one reason for advancing the 5-amino-1-(carbamoylmethyl)-1H-1,2,3-triazole-4-carboxamide. The fact that substitutions that likely perturb the conformational dynamics, such as N-methylation of the amide bonds, is consistent with this hypothesis. Nonetheless, the exact target of lead **1** or analog **14** awaits elucidation through structural or mutational studies and allosteric inhibition may well be the mechanism of action due to the inaccessibility of the active site to all but its natural substrate (Culyba et al., 2015).

One likely driving force for the frequent use of this scaffold in varied therapeutic applications is its low cytotoxicity, as evidenced by the CC_50_ values of 277 μM and > 500 μM. Other properties of **14** also indicate that it is a promising starting point, although ongoing optimization is needed. The properties of the molecule fall within Lipinski’s rules for drug-likeness(Lipinski et al., 1997): it has a molecular weight of 380.4 (<500), three hydrogen bond donors (≤5), six hydrogen bond acceptors (≤10) and a cLogP of 1.63 (≤5). In comparison to oral drugs for non-infectious diseases, antibacterial compounds tend to have greater polarity (O’Shea and Moser, 2008; Brown et al., 2014) which provides better solubility (useful for IV drugs) and may enable improved permeability through the outer membrane of Gram-negative bacteria (Nikaido, 2003; Brown et al., 2014). Low lipophilicity is also preferred to avoid off-target activities and cytotoxicity (Livi et al., 1979). Compound **14** has a polar surface area of 124 Å^2^ which is below the value of 140 Å^2^ above which permeability is typically an issue. These properties define **14** as a drug-like small molecule modulator of the SOS response.

For small molecule SOS modulators to prove useful to address therapeutic challenges, there are two important features of the molecules which will be necessary. First, the molecules must have sufficient breadth to allow for their use against multiple potential pathogens. Although our initial lead **1** showed only limited reactivity against LexA from *P. aeruginosa* (Figure 5), our optimization around the scaffold encouragingly revealed **14** as an analog with similar potency against LexA from *E. coli* and *P. aeruginosa*. This development is important because pathogens such as *P. aeruginosa* are associated with chronic infections. Frequent antibiotic exposure in patients with cystic fibrosis or other immunocompromising conditions make the risks of acquired resistance particularly high in these patients. In addition to cross-species reactivity, small molecule modulators must also show sufficient potency *in vivo*. The improved analog **14** shows SOS inhibition activity using the GFP reporter assay in the efflux compromised Δ*tolC E. coli* strain. Encouragingly, at high concentrations, **14** also reduced the rate of ciprofloxacin-induced mutation (Figure 4). Although these activities against *E. coli* are promising, these results suggest that the potency of the current leads requires additional improvement, especially because genetic studies not only suggest that potent SOS inhibition is necessary to fully potentiate antibiotic effects but also reveal that mutation rates can be reduced even further (Mo et al., 2016).

The trigger for the activation of the SOS response is genotoxic stress which many antibiotics induce. Molecules that attenuate the activation of the SOS response could therefore reduce the ability of pathogens to adapt and evolve under antimicrobial treatment. Evidence suggests that such a therapeutic would be most effective when used as an adjuvant to an antibiotic whose mechanism of action involves directly damaging DNA, e.g., fluoroquinolones (Mo et al., 2016). The improvements in potency and cross-species activity with **14** suggest that although ongoing work is needed to improve existing leads, discovery of such a therapeutic DISARMER is a feasible pursuit. Combining fluoroquinolones with a potent DISARMER could provide advantages similar to those that β-lactamase inhibitors have provided for β-lactam antibiotic therapy. These possible advantages include extension of the useful lifetime of an antibiotic, increased susceptibility of bacteria to antibiotics, and slowed acquisition of resistance, all of which offer alternative strategies to address the challenges posed by bacterial pathogens.

## Author Contributions

TS, RK, AR, and SB designed the experiments. TS, BL, CM, MC, and ZH performed the experiments. TS, RK, and SB analyzed the data. TS, RK, and SB wrote the manuscript. All authors reviewed and edited the manuscript.

## Conflict of Interest Statement

The authors declare that the research was conducted in the absence of any commercial or financial relationships that could be construed as a potential conflict of interest.
