# Supplementary material for: Advancement of the 5-Amino-1-(Carbamoylmethyl)-1H-1,2,3-Triazole-4-Carboxamide Scaffold to Disarm the Bacterial SOS Response
Source: Front Microbiol. 2018 Dec 18;9:2961. doi: 10.3389/fmicb.2018.02961 (PMC6305444; doi:10.3389/fmicb.2018.02961)
Supplement: Supplementary file 1 [file Data_Sheet_1.pdf]

Advancement of the 5-amino-1-(carbamoylmethyl)-1H-1,2,3-triazole-4-carboxamide scaffold to disarm the bacterial SOS response

Trevor Selwood[1,2], Brian J. Larsen[3], Charlie Y. Mo[1,2], Matthew J. Culyba[1,2], Zachary M. Hostetler[1,2], Rahul M. Kohli*[,1,2], Allen B. Reitz[3], Simon D.P. Baugh*[,3]

[1] Department of Medicine, University of Pennsylvania, Philadelphia, PA, 19104
[2] Department of Biochemistry and Biophysics, University of Pennsylvania, Philadelphia, PA, 19104
[3] Fox Chase Chemical Diversity Center, Inc., Doylestown, PA, 18902

<u>Supplementary Information</u>

Supplementary Methods	Alternative synthetic routes
Supplementary Figure 1	Representative titrations curves from HTS
Supplementary Figure 2	Binding of **14** to LexA alone
Supplementary Figure 3	LexA DNA binding activity is not impacted by **14**
Supplementary Figure 4	Cell size is not impacted by **14**
Supplementary Table 1	Alternative lead analogs synthesis and inhibition

SUPPLEMENTARY METHODS

Alternative synthetic routes

Synthesis D: A mixture of acetylene component (0.25 mmol) and azido component (0.275 mmol) in THF (1.25 mL) was treated with CuI (0.025 mmol) and diisopropylethylamine (0.50 mmol). The reaction was stirred at 25°C for 60 hours before treatment with dimethylacetamide (1.25 mL). The mixture was heated in a microwave reactor at 120°C for 10 minutes and at 170°C for 15 minutes before concentration. The resulting residue was purified via column on silica (0-100% ethyl acetate: hexane), to give the desired product.

Synthesis E: A mixture of acetylene component (0.2 mmol) and azido component (0.2 mmol) in dimethylacetamide (1 mL) was heated in a microwave reactor at 150°C for 11 minutes. The reaction was treated with water (1 mL), filtered and the filtrate was purified via reverse phase HPLC using acetonitrile in deionized H_2O (with 0.1% TFA in both solvents). Following elution, the solvent was removed by evaporation and lyophilization to yield the desired product.

Synthesis F: A mixture of aminotriazole component (0.025 mmol) in DMF (0.24 mL) was cooled to 0°C, before addition of diisopropylethylamine (0.049 mmol) and acetyl chloride (0.043 mmol). The reaction was stirred for 4 hours before 4-dimethylaminopyridine (DMAP) (0.087 mmol), diisopropylethylamine (0.049 mmol), and acetyl chloride (0.1 mmol) were added. The resulting mixture was stirred at 20°C for 16 hours before the temperature was raised 35°C and maintained for a further 24 hours. The mixture was treated with DMF (0.15 mL) and purified via reverse phase HPLC using acetonitrile in deionized H_2O (with 0.1% TFA in both solvents). Following elution, the solvent was removed by evaporation and lyophilization to yield the desired product.

LexA-only orthogonal ^{32}P-LexA cleavage assay

The buffer and protein concentrations were the same as the for the orthogonal ^{32}P-LexA cleavage assay except for the absence of RecA and the time and temperature of the incubation. Buffer alone replaced the addition of RecA in buffer and incubations were performed for 4 hours at 37˚C.

Thermal shift assay

Assays were performed in a MicroAmp optical 96-well reaction plate (Applied Biosystems) was used to analyze 21 µL sample solutions. Each well contained 5 µM unlabeled full length *E. coli* LexA with the PKA phosphorylation site and 1x SYPRO Orange (Molecular Probes) in 0.1 M Tris, pH 6.5, 150 mM NaCl, 5 mM MgCl2, 0.1 mM TCEP, 5% DMSO with or without 50 µM **14**. The plate was sealed with optical adhesive film (Applied Biosystems) and a 7500HT Fast Real-Time PCR System (Applied Biosystems) was used to heat the plate from 20 °C to 95 °C in 0.1 °C increments. Changes in fluorescence were monitored and the data were fitted using equation 1 using Igor Pro (WaveMetrics).

$$FI(T) = FI_{pre} + \frac{(FI_{post} - FI_{pre})}{1 + exp^{(\frac{T_m - T}{Slope})}} \qquad \text{(Eqn S1)}$$

In equation S1 FI(T) is the measured fluorescence intensity at temperature T; FI_{pre} and FI_{post} are the pre-transitional and post-transitional fluorescence intensities, respectively; T_m is the temperature midpoint for the protein unfolding transition; and slope is the slope factor.

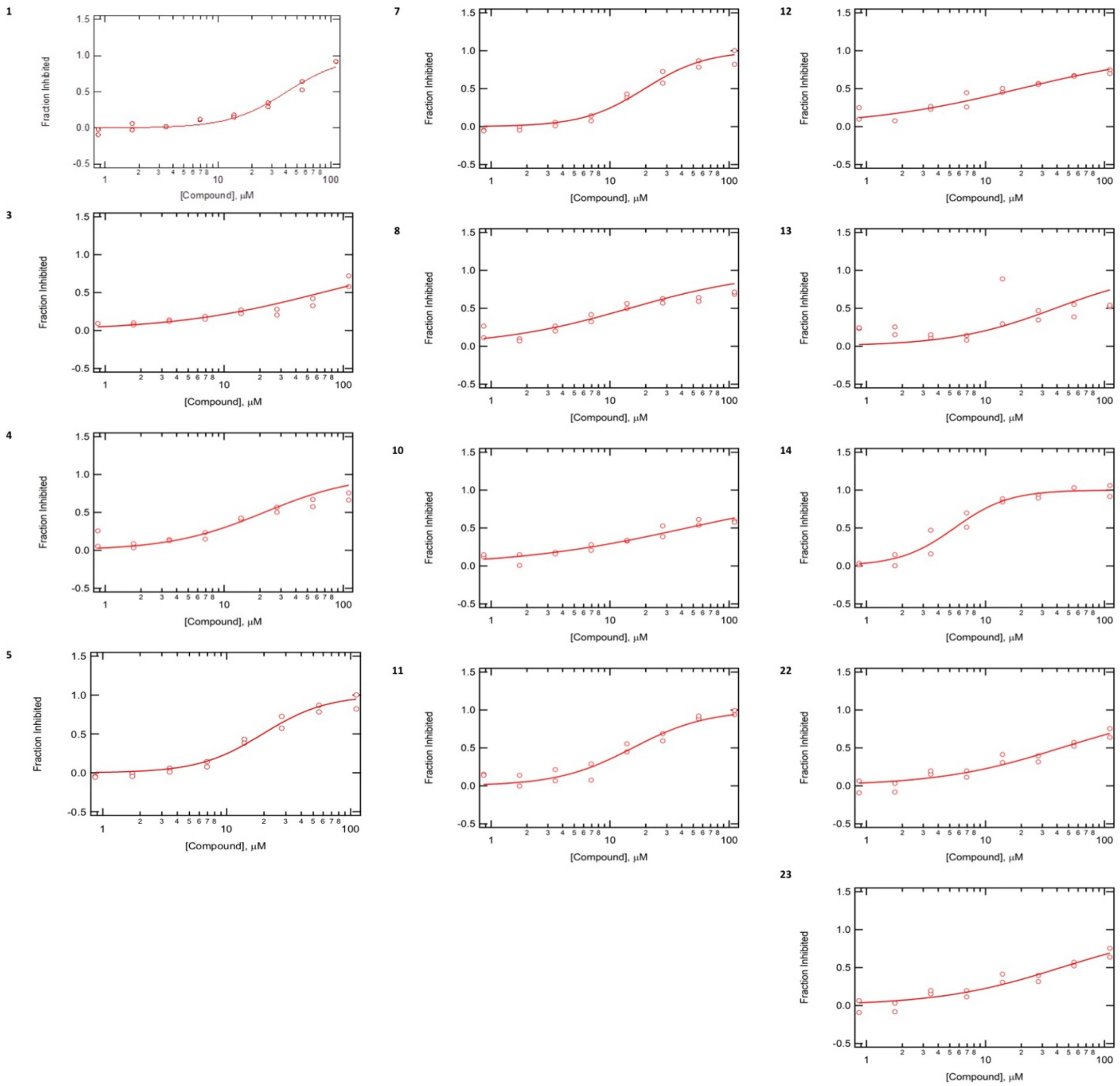

Supplementary Figure 1. Representative titration curves from high throughput screening.

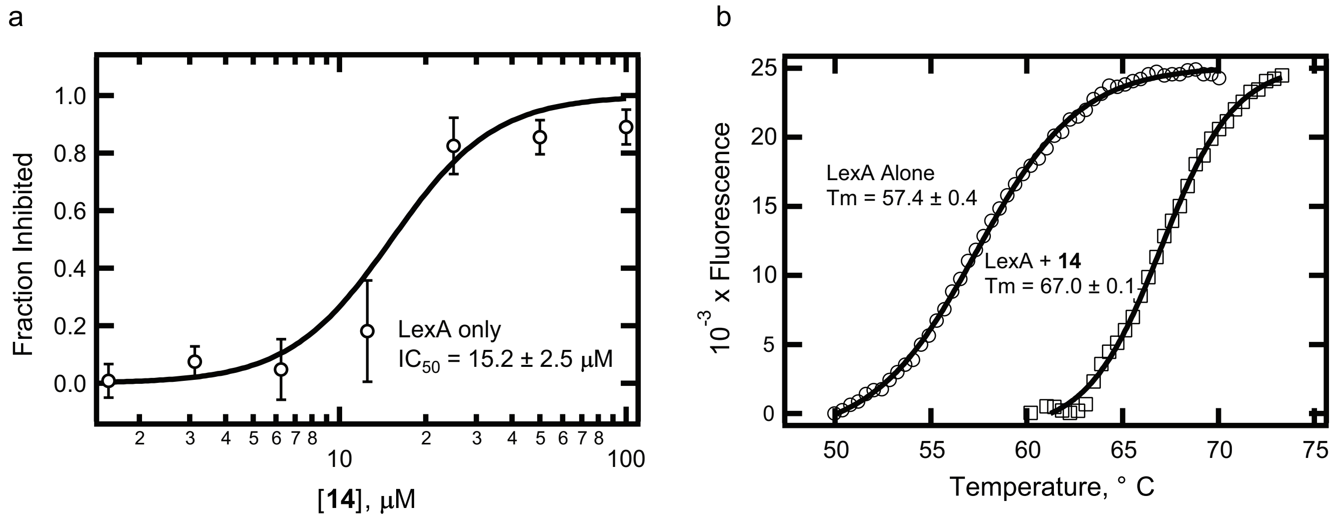

Supplementary Figure 2. Binding of **14** to LexA alone. (a) Inhibition of the auto-proteolysis of ^{32}P-labelleled full length *E. coli* LexA by **14** in the absence of RecA. All data represent the mean of two independent measurements and the error bars represent the range of error. The error of the IC_{50} value is the standard deviation of the fit of equation 1 to the data. (b) Thermal shift observed upon binding of **14** to full length *E. coli* LexA. The concentrations of LexA and **14** were 5 μM and 50 μM, respectively. The errors for the Tm values are the standard deviations of the fits of equation S1 to the data.

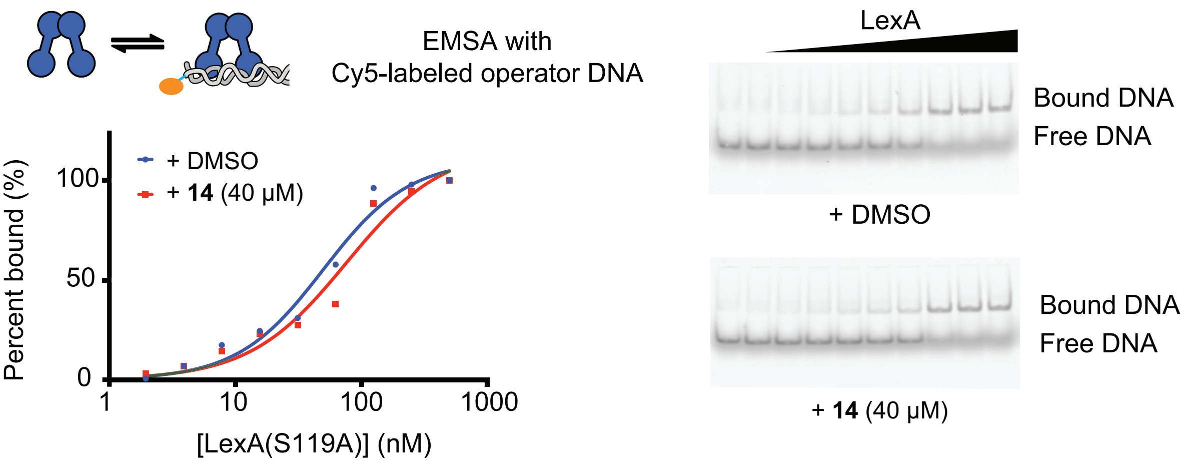

Supplementary Figure 3. LexA DNA binding activity is not impacted by **14**. Varying concentrations of catalytically inactive LexA(S119) were incubated with Cy5-labeled DNA containing an LexA binding sequence (SOS box). The titrations were performed in the presence of DMSO or 40 μM of **14**. The samples were separated on a non-denaturing polyacrylamide gel (at right) and the bound fraction calculated.

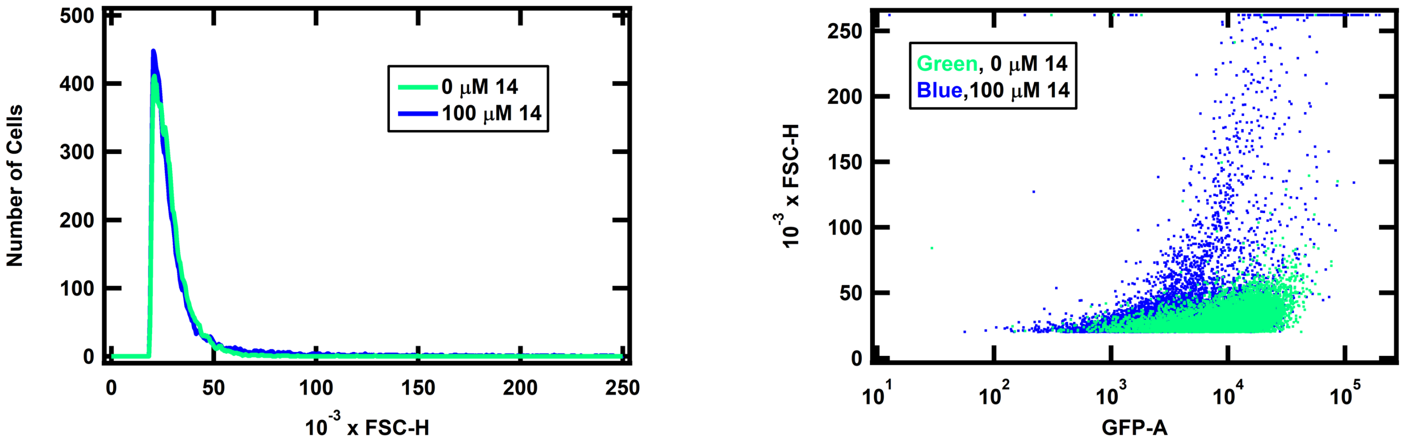

Supplementary Figure 4. Cell size is not impacted by **14**. At right is a dot plot for *E. coli* MG1655 Δ*sulA* Δ*tolC* showing the GFP fluorescence and the side-scatter, which reflects cell size. The reduction in GFP fluorescence can be observed on the x-axis in the presence of **14**, relative to its absence. These cells are not expected to filament because of the absence of SulA, however a minor population appears which is likely some cellular aggregates in the presence of **14**. Nonetheless, this is a very minor population as evidenced by the histogram plot shown at left, where the vast majority of cells are similarly sized, despite the clear reduction in GFP.

Supplementary Table 1. Alternative lead analog synthesis and inhibition

Compound	Structure	*E. coli* IC_{50}/μM[1]	Synthesis[2]	Yield, %
**17**	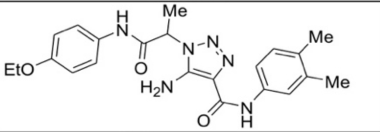	>100	C	9
**18**	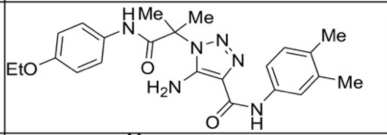	>100	C	5
**19**	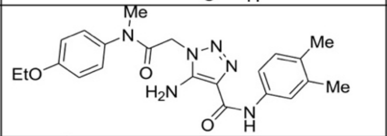	>100	C	8
**20**	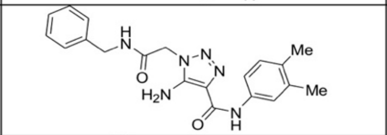	>100	A	24
**21**	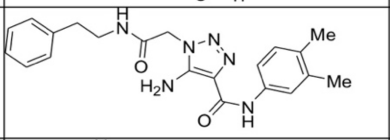	>100	A	66
**22**	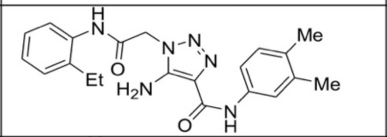	43 ± 6	n/a	commercial
**23**	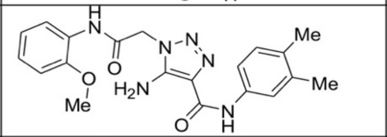	55 ± 7	n/a	commercial
**24**	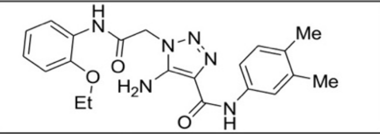	>100	n/a	commercial
**25**	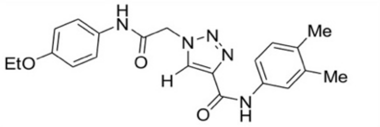	>100	D	22
**26**	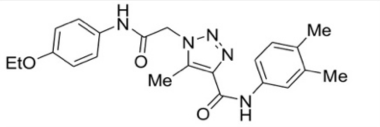	>100	D	12
**27**	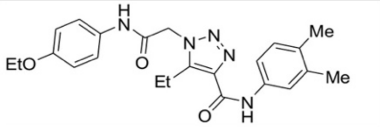	>100	E	7

Supplementary Table 1. Alternative lead analog synthesis and inhibition (continued)

**28**	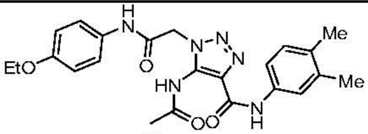	42 ± 10	F	50
**29**	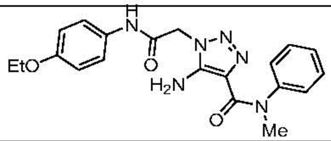	>100	C	43
**30**	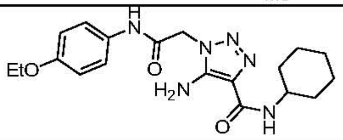	>100	A	24
**31**	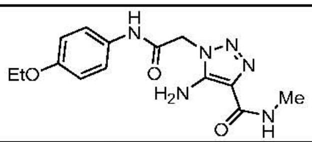	>100	C	11
**32**	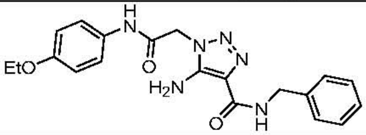	>100	A	25
**33**	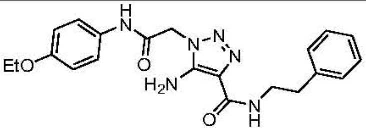	>100	A	30

[1] IC50 values are the avearge of 4 determinations and the errors are ± 1 SD.

[2] A- NaOEt B- Cs_2CO_3 C- NaOMe D- DIPEA E- heat F- acetyl chloride
